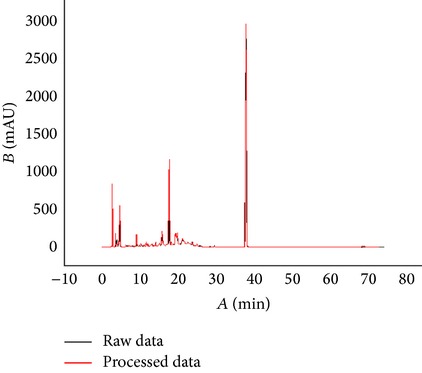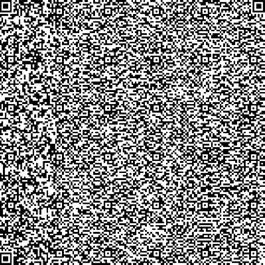# Traceability and Quality Control in Traditional Chinese Medicine: From Chemical Fingerprint to Two-Dimensional Barcode

**DOI:** 10.1155/2015/251304

**Published:** 2015-05-18

**Authors:** Yong Cai, Xiwen Li, Mei Li, Xiaojia Chen, Hao Hu, Jingyun Ni, Yitao Wang

**Affiliations:** ^1^State Key Laboratory of Quality Research in Chinese Medicine, Institute of Chinese Medical Sciences, University of Macau, Macau; ^2^Information Technology College of Beijing Normal University, Zhuhai Campus, Zhuhai 519087, China; ^3^Research Center for Pharmacognosy, Institute of Chinese Materia Medica, China Academy of Chinese Medical Sciences, Beijing 100700, China

## Abstract

Chemical fingerprinting is currently a widely used tool that enables rapid and accurate quality evaluation of Traditional Chinese Medicine (TCM). However, chemical fingerprints are not amenable to information storage, recognition, and retrieval, which limit their use in Chinese medicine traceability. In this study, samples of three kinds of Chinese medicines were randomly selected and chemical fingerprints were then constructed by using high performance liquid chromatography. Based on chemical data, the process of converting the TCM chemical fingerprint into two-dimensional code is presented; preprocess and filtering algorithm are also proposed aiming at standardizing the large amount of original raw data. In order to know which type of two-dimensional code (2D) is suitable for storing data of chemical fingerprints, current popular types of 2D codes are analyzed and compared. Results show that QR Code is suitable for recording the TCM chemical fingerprint. The fingerprint information of TCM can be converted into data format that can be stored as 2D code for traceability and quality control.

## 1. Introduction

The industrial chain of traditional Chinese medicine (TCM), from the production of raw materials to the sale of their finished products, is a complicated multilink process. It is an absolutely important task to ensure the quality of TCM to be safe, effective, stable, and controllable in the whole process. Although the advent of modern analytical technologies made a positive impact on component determination [1–3] and undoubtedly will continue to make a substantial contribution to quality control, current quality test in each part of the process is relatively independent and testing results can hardly be shared, which bring regulatory blind spots and difficulty of quality traceability. None of the available tools can combine all links and work for TCM traceability in the whole production process. Current traceability technologies included radio frequency identification [4–6], barcode [7], and other combined techniques based on web [8]. However, these technologies were mainly used in circulation links and they could not provide quality information of products. A new method is required to run through each link of TCM and can carry quality traceability information.

Chemical fingerprinting was a comprehensive and quantified testing method. It can be constructed based on the systematic research on the chemical constituents for the evaluation of authentication [9, 10], reliability [11, 12], and stability [13–15] of TCM and their semifinished products, and it has now become a main method for quality determination and was accepted in Chinese Pharmacopoeia. However chemical fingerprints cannot be directly applied to Chinese medicine traceability due to several limitations. First, chemical fingerprints are stored in image format which has a large data capacity. It is difficult to be compressed and exported for a batch of information management. Second, quality information cannot be obtained through direct scanning of chemical fingerprints. It is not easy to transport quality information and the information cannot easily be shared between different links in the process of the production and the circulation of TCM.

Barcode technology is an important tool for modern logistics management due to its ability in quick and accurate information extraction and batch management. It has been widely used in the manufacturing [7, 8, 16], medication administration [17–19], and retailing industries [20, 21]. It was recently reported that DNA barcoding could be used for traceability [22]. The DNA molecular sequence cannot be automatically recognized by direct scanning, and the large size of printout is inconvenient to the application of distribution management; researchers conducted studies on converting DNA sequences into 2D barcode [23–25]. Chemical fingerprints share the similar vector features with the molecule sequence and the fingerprint, but so far no literature can be found on converting TCM fingerprint into 2D barcode. In this paper, with the high performance liquid chromatography (HPLC) technology, the chemical fingerprints were constructed based on the quality testing results and chemical quality data were extracted. After data normalization, the fingerprint information can be converted into the data format that can be stored by barcode and then further converted into the 2D barcode of TCM fingerprint. By comparing different encoding types, QR Code is finally recommended as the best barcode for quality traceability.

## 2. Materials and Methods

### 2.1. Construction of Chemical Fingerprints

Three Chinese medicines were randomly selected and their chemical fingerprints were constructed using HLPC technology (YinYangHuo and RouCongRong refer to [26, 27], resp.). The detailed method of MuDanPi detection was as follows: a Zorbax SB-C18 column (250 × 4.6 mm I.D., 5 *μ*m) with a Zorbax SB-C18 guard column (12.5 × 4.6 mm I.D., 5 *μ*m) was used. The samples were separated using a gradient mobile phase consisting of 0.5% acetic acid (A) and acetonitrile (B). The gradient condition is 0–40 min, 10%–50% B; 40–60 min, 50%–100% B; 60–65 min, 100% B. The separation was performed on an Agilent series 1200 liquid chromatography (Agilent Technologies, Santa Clara, CA, USA), equipped with a vacuum degasser, a quaternary pump, an autosampler, and a diode array detector (DAD).

### 2.2. Data Analysis

**Figure 1:**
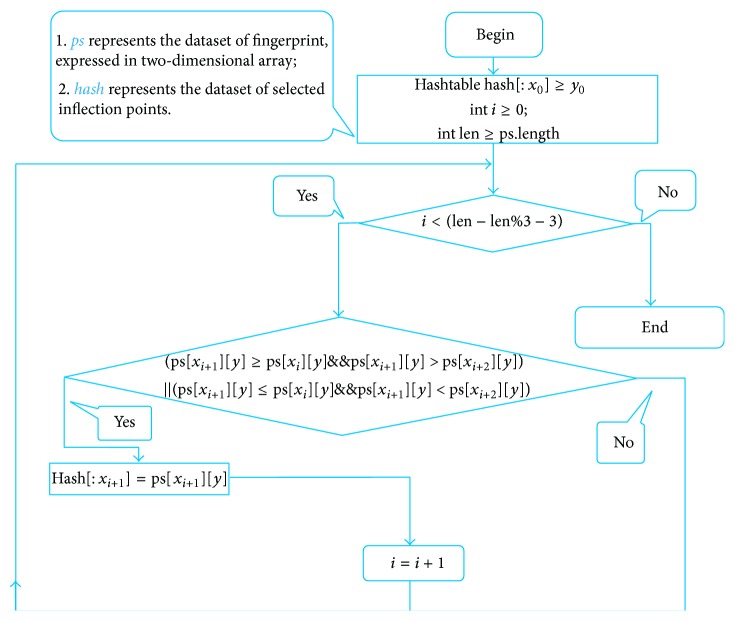
Program for selecting inflexion points.

DIF (or TXT, CSV) data (see File S1 of the Supplementary Material available online at http://dx.doi.org/10.1155/2014/251304) were downloaded from HPLC instrument and were saved as excel files. These files were processed for data standardization: remove negative time and absorbance data; find inflexion points in chemical fingerprints; keep one decimal fraction of time data and remove duplicate ones; cut some redundant no-absorbance data. A software program under the environment of Ruby (Netbeans IDE 6.5, Ruby 1.8.7, Gem spreadsheet 0.9.5) was created to carry out data filtering, and the detailed process can be referred to Figure 1. The processed data would then be saved as the format of text and excel (see Files S2 and S3). The text format will be strung for converting into 2D code, while the excel format will be used to convert into chemical fingerprints.

### 2.3. Selection of Barcode Type and Evaluation of Processed Data

In order to select suitable barcode types for storing data from chemical fingerprints, different 2D barcode types were compared in the aspect of encoding features. Filtered data stored in the excel file were inputted to liquid chromatograph and reanalyzed peak areas to evaluate whether data treatment has a significant influence on corresponding chemical composition content. OriginPro software was used to regenerate the TCM fingerprints. QR Code Generators were used to output 2D barcodes online (http://qrgenerator.qrcreator.net) and the parameters involved are listed as follows: ECC (Error Correcting Code): L-smallest; Size: 2.

## 3. Results and Discussion

With the development of modern industry of Chinese medicine, demand for Chinese medicinal herbs has grown rapidly over past decades. Although the HPLC technology can make a positive impact on quality control, quality traceability is still hard to implement because chemical fingerprints cannot be easily transported. This study successfully created a way to convert chemical information to barcodes for TCM quality traceability.

Based on the analysis of the test data of three different TCM chemical fingerprints (Figure 1), it was found that the sampling frequency was 0.4 seconds, and the sizes range of dataset was from 200 kilobytes to 370 kilobytes. However, all kinds of barcodes available cannot store such huge data of chemical fingerprints and therefore data filtering is required. After data filtering using Ruby program, the data sizes were from 0.7 kilobyte to 2.8 kilobyte and their main content was numeric characters. Compared to the original data, the compression ratio in terms of length of string is between 0.23% and 0.64%, ranging from 713 to 2323, while the compression ratio in terms of data point is between 1.12% and 2.99%, ranging from 110 to 337 (Table 2). With the algorithm (Figure 1), the dataset of the key inflexion point of the original fingerprint was retained and the data amount is hugely reduced, which created conditions for the generation of 2D barcode. The result from the above experiment shows that the curve after processing was fitted highly with the curve before processing; the main peak time and the peak delay are basically the same (Table 3). In order to test whether data processing has an influence on peak area of chemical fingerprint, we made a comparison between the two peak areas of raw data and processed data. Results showed that there was no significant difference (*P* < 0.05) between them (Table 3), which demonstrated that data standardization in this study can be used for converting chemical information to barcodes for quality traceability.

Barcode has two encoding types, one-dimensional code and two-dimensional code (Tables S1 and S2). The former one can be read with a fast speed and is more commonly used in retailing industries or manufacturing such as commodity packaging and electronic tickets. However one-dimensional code has a smaller data storage capacity (20 bytes) and cannot be used to hold bigger data. The latter one can store more information (2000 bytes) including text, images, fingerprint, and signature in limited space and can be used without the computer. Two-dimensional code becomes more and more popular in recent years. It was found that the size of chemical fingerprint data after filtering was still beyond 2 kilobytes. Such big data are difficult to be converted into barcode directly and they are difficult to be read. In addition, most 2D barcodes can store less than 2 kilobytes. Therefore, the capacity and the compression ratio become the most important filtering criteria in the choice of barcode type. Among all 2D barcodes, Data Matrix, Aztec Code, QR Code, Vericode, PDF 417, PDF 417 Truncated, Codablock F, and Code One meet the criteria. With further analysis to the above eight types of 2D barcodes, QR Code, Data Matrix, and PDF 417 are the most appropriate ones for storing numeric character. Comparative results (Table 1) show that QR Code is far more appropriate than the other two types in terms of storage capacity, identification speed, and readable direction. In addition, reconstructed chemical fingerprints from processed data recommended QR Code as the best 2D barcode for TCM quality traceability (Table 3).

## 4. Conclusion

The production chain of traditional Chinese medicine includes multiple links. Traceability is very important for guaranteeing TCM quality. This study takes the first step in the combination between quality detection and traceability. It was demonstrated that the data from TCM chemical fingerprints can be converted into 2D barcodes which can be used in the whole TCM industrial process. Similarly, the conversion from data of UV, IR, MS, NMR, and TLC to 2D barcode is also a potential topic for further traceability study. There is further space for improvement of data filtering. TCM fingerprints from different kinds of detecting devices require data preprocessing. The optimal data algorithm depends on whether the processed data is consistent with the original data and how much space will be required.

## Supplementary Material

Supplementary Table S1: shows the main difference between the one dimension and two-dimensional code.
Supplementary Table S2: shows a total of 53 types of one dimension codes and 14 types of two-dimensional codes tested in this study.

## Figures and Tables

**Table 1:** Comparison between different two-dimensional barcodes.

Code system	QR Code	Data matrix	PDF 417
Developer	DENSO (Japan)	RVSI Acuity CiMatrix (USA)	Symbol Technologies Inc. (USA)
Type	Matrix	Matrix	Stacked barcode
Data capacity			
Numeric	7,089	3,116	2,710
Alphanumeric	4,296	2,355	1,850
Binary	2,953	1,556	1,018
Kanji	1,817	778	554
Error correction level	Max 30%	Max 25%	Max 50%
Identification speed	30/s	2~3/s	3/s
Readable direction	360°	360°	+/−10°
Main features	Large capacity Small printout size	Small printout size	Large capacity
Main usages	All categories	FA	OA
Standardization	AIM International JIS, ISO	AIM International ISO	AIM International ISO
Sample picture	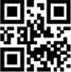	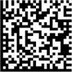	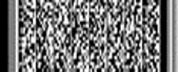

**Table 2:** Changes of length of string and data points during data filtering process.

Sample	Step 1 bytes/points	Step 2 bytes/points	Step 3 bytes/points	Compression ratio
YinYangHuo	277508/9001	57311/8320	1228/185	0.44%/2.05%
Roucongrong	306359/9751	16581/2481	713/110	0.23%/1.12%
MUDANPI	359105/11250	53758/7328	2108/291	0.58%/2.58%

Step 1: download raw data from liquid chromatograph; step 2: remove negative data; step 3: find inflexion points and keep one decimal fraction of time data, remove duplicate time data, and redundant no-absorbance data.

**Table 3:** Comparison of chemical fingerprints between raw data and processed data and generated QR Codes.

Sample	Chemical fingerprints from raw data and processed data	QR Code
YinYanghuo	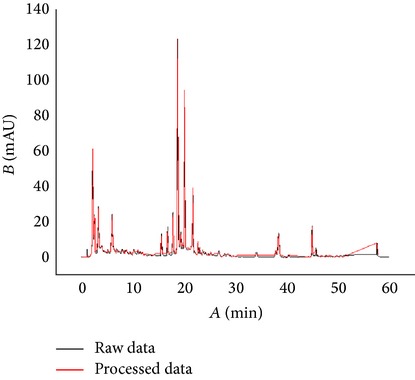	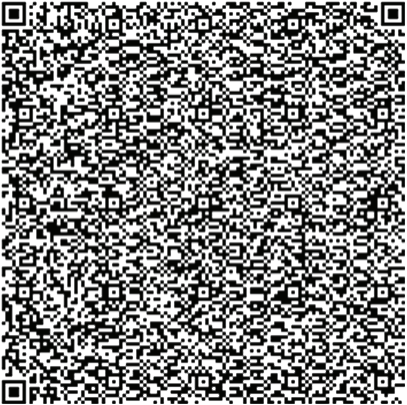

Roucongrong	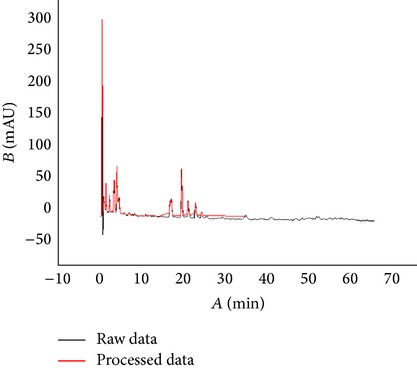	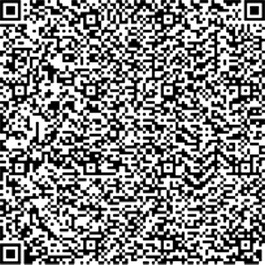

Mudanpi